# Producing Proofs of Unsatisfiability with Distributed Clause-Sharing SAT Solvers

**DOI:** 10.1007/s10817-025-09725-w

**Published:** 2025-05-27

**Authors:** Dawn Michaelson, Dominik Schreiber, Marijn J. H. Heule, Benjamin Kiesl-Reiter, Michael W. Whalen

**Affiliations:** 1 Amazon Web Services, Seattle, WA USA; 2 Karlsruhe Institute of Technology, Karlsruhe, Germany; 3 Carnegie Mellon University, Pittsburgh, PA USA; 4 Amazon Web Services, Munich, Germany; 5 University of Minnesota, Minneapolis, MN USA

**Keywords:** SAT solving, Proofs, Distributed computing, Cloud computing, HPC

## Abstract

Distributed clause-sharing SAT solvers can solve challenging problems hundreds of times faster than sequential SAT solvers by sharing derived information among multiple sequential solvers. Unlike sequential solvers, however, distributed solvers have not been able to produce proofs of unsatisfiability in a scalable manner, which limits their use in critical applications. In this work, we present a method to produce unsatisfiability proofs for distributed SAT solvers by combining the partial proofs produced by each sequential solver into a single, linear proof. We first describe a simple sequential algorithm and then present a fully distributed algorithm for proof composition, which is substantially more scalable and general than prior works. Our empirical evaluation with over 1500 solver threads shows that our distributed approach allows proof composition and checking within around 3$\times$ its own (highly competitive) solving time.

## Introduction

SAT solvers are general-purpose tools for solving complex computational problems. By encoding domain problems into propositional logic, users have successfully applied SAT solvers in a plethora of relevant fields such as formal verification [49], electronic design automation [33], and mathematics [12]. The list of applications has grown significantly over the years, mainly because algorithmic improvements have led to orders of magnitude improvement in the performance of the best sequential solvers [9].

Despite all this progress, there are still many problems that cannot be solved quickly with even the best sequential solvers, pushing researchers to explore ways of parallelizing SAT solving. One approach that has worked well for specific problem instances is *Cube-and-Conquer* [25, 28], which can achieve near-linear speedups for thousands of cores but requires domain knowledge about how effectively to split a problem into subproblems. An alternative approach that does not require such knowledge is *clause-sharing portfolio solving* [21], which has recently led to distributed solvers [44] achieving impressive speedups (e.g., 40–400$\times$ at 3000 cores, depending on problem difficulty [45]) over the best sequential solvers across broad sets of benchmarks [19].

Today, distributed clause-sharing SAT solvers are some of the most powerful tools available for solving hard SAT problems. However, there is an important caveat: unlike sequential solvers, current distributed clause-sharing solvers cannot produce proofs of unsatisfiability. A direct consequence is that these distributed solvers cannot be used for proving theorems [23, 28, 46]. Even in cases where proofs are not strictly required, it is important to be able to trust the output of an algorithm.^1^ For instance, in bounded model checking [14]—a crucial verification tool and one of the most prominent applications of SAT solving—a formula’s reported unsatisfiability is interpreted as a system *behaving correctly* up to the considered bound. Therefore, the safety and reliability of crucial systems may depend on a SAT solver answering correctly.

In this sense, we argue that distributed clause-sharing solvers are lacking compared to sequential SAT solvers in terms of general trustworthiness. The latter, while complex pieces of software, not only generate verifiable proofs but are also being rigorously tested (e.g., [6, 13]) and feature limited external interfaces. Distributed clause-sharing solvers, on the other hand,

- are more costly (and thus more difficult) to test rigorously;
- make use of several different SAT solvers configured in many different ways [3];
- run many execution threads concurrently; and
- make use of non-trivial interfaces for data transfer such as message passing [3] and inter-process communication [44].

All of these properties have some potential to introduce faults or correctness issues in certain corner cases, which makes it all the more critical to be able to verify the system’s output independently.

Although there has been foundational work in producing proofs for shared-memory clause-sharing SAT solvers [7, 27], existing approaches are not general enough for large-scale distributed portfolio solvers. In this work, we address this issue and present the first scalable approach for generating proofs for such solvers. To construct proofs, we maintain *provenance* information about shared clauses in order to track how they are used in the global solving process, and we use the recently-developed LRAT proof format [16] to track dependencies among partial proofs produced by solver instances. By exploiting these dependencies, we are then able to reconstruct a single linear proof from all the partial proofs produced by the sequential solvers. We first outline a simple sequential algorithm for proof reconstruction before devising a parallel algorithm that we implement in a fully distributed way. Both algorithms produce independently-verifiable proofs in the LRAT format. We demonstrate our approaches using an LRAT-producing version of the sequential SAT solver CaDiCaL [8] to turn it into a clause-sharing solver, and then modify MallobSat to orchestrate a portfolio of such CaDiCaL instances while tracking the IDs of all shared clauses.

We evaluate our approaches from the perspective of efficiency, benchmarking the performance of our clause-sharing portfolio solver against the winners of the cloud, parallel, and sequential tracks from ISC 2022. Our approach introduces overhead in terms of solving, proof reconstruction, and proof checking. We examine this overhead in detail and show that our approach is still considerably faster than sequential approaches. We also demonstrate that our approach substantially outperforms prior work on proof production for clause-sharing portfolios [27].

Finally, we discuss the latest advances in this area. Most notably, our initial work served as a motivation for Pollitt et al. to implement a sequential solver with full, efficient LRAT support [37]. Integrating this solver and dropping the previously required pre- and postprocessing stages now results in even better scaling behavior.

**Context** The article at hand is a significantly extended version of our TACAS 2023 conference publication [35]. We integrated supplementary content from the corresponding author’s dissertation [42], notably a proof of correctness for our distributed proof production approach and a more detailed analysis of experiments. Furthermore, we describe important recent developments in this topic and are able to present significantly improved results in follow-up experiments. Since these latest results complement our original results, which feature a less developed setup but more competitors and analyses, we include both of them as important stages in our line of work.

This article is structured as follows. In Sect. 2, we provide the relevant background for our discussion. In Sect. 3, we outline the general problem of producing proofs for distributed SAT solving and a first sequential algorithm for proof combination. In Sect. 4, we describe a more general algorithm for distributed-memory setups. We discuss our original implementation in Sect. 5 and the according empirical evaluation in Sect. 6, then we describe and assess our latest advances on the topic in Sect. 7. We conclude with a summary and an outlook in Sect. 8.

## Background

In this section, we introduce relevant preliminaries for the paper, including SAT, proofs of unsatisfiability, and parallel and distributed SAT solving.

### The SAT Problem

A *Boolean variable* can only be true or false. A *literal* is a Boolean variable or its negation. A *clause* is a disjunction of literals, i.e., a logical expression that evaluates to true if and only if at least one of the literals in the clause is true. A *Conjunctive Normal Form* (CNF) formula is a conjunction of clauses, i.e., a logical expression that evaluates to true if and only if each of the clauses evaluates to true.

An *assignment* $\alpha$ for a logical expression *F* assigns values to some of the variables occurring in *F*. $\alpha$ is *partial* if some variables in *F* are left unassigned, and $\alpha$ is *total* otherwise. If $\alpha$ is total and *F* evaluates to true under $\alpha$, then we write $\alpha \models F$ and say that $\alpha$ *is a model for* *F* or that $\alpha$ *satisfies* *F*. We refer to such an assignment as a *satisfying assignment (for F)*. A CNF formula *F* is *satisfiable* if and only if a satisfying assignment to *F* exists. Otherwise, *F* is *unsatisfiable*.

An instance of the *SAT decision problem* is given as a CNF formula *F*. The task is to decide whether *F* is satisfiable. A common extension of the SAT decision problem, which we refer to as the *constructive SAT problem*, additionally requires outputting a satisfying assignment $\alpha$ if *F* was found to be satisfiable. Likewise, we consider the *certified SAT problem* as an extension of the constructive SAT problem which additionally requires outputting an *unsatisfiability certificate* ${\mathcal {C}}$ if *F* was found to be unsatisfiable. Intuitively, an unsatisfiability certificate is a chain of logical reasoning that the solver used to derive unsatisfiability and that others can verify independently.

Today’s most efficient sequential SAT solvers commonly build upon the *Conflict-Driven Clause Learning* (CDCL) approach [34]. Intuitively, the solver carefully searches the space of partial variable assignments and derives *conflict clauses* from encountered logical conflicts. These clauses can be useful to prune the search for a satisfying assignment on the one hand and to derive the *empty clause*, demonstrating unsatisfiability, on the other hand. Maintaining and garbage-collecting learned clauses in a sensible manner is an important line of research in SAT solving [1, 36].

### Proofs of Unsatisfiability

In contrast to the pure decision problem, the certified SAT problem requires a justification for the produced result. For the satisfiable case, this is straightforward. All common SAT solving approaches conclude the satisfiability of a formula by constructing a satisfying assignment. This assignment serves as a justification since it can be verified in linear time by evaluating the formula on the assignment. For the unsatisfiable case, the usual justification is the solver’s chain of logical reasoning leading to the *empty clause*, which demonstrates unsatisfiability. This chain is not necessarily linear in the problem input, and, in fact, certain inputs require an exponentially-sized proof when using common reasoning techniques [20, 24].

Consider a formula *F* and a sequence of clauses $C := \langle c_1, c_2, \ldots , c_n \rangle$ learned by a CDCL solver *S* while processing *F*. The last clause $c_n$ is the empty clause, i.e., the solver has derived unsatisfiability for *F*. In order to verify that the result is correct, we can check for each $i \in \{1, \ldots , n\}$ if $c_i$ is indeed a logical implication of the prior formula:

$$\begin{aligned} \left( F \cup \bigcup _{j=1}^{i-1} c_j\right) {\mathop {\Rightarrow }\limits ^{?}} c_i \end{aligned}$$

Many clause learning and preprocessing techniques have a convenient property named the *Reverse Unit Propagation* (RUP) property [48]. For any clause found with the RUP property, the check above can always be achieved by means of unit propagation: We assert each literal of $c_i$ to be false and then check if unit propagation leads to a direct conflict. In this case, we showed that $F \wedge \lnot c_i$ is unsatisfiable, hence $c_i$ is a logical consequence of *F* and *S* was correct to derive it. If no conflict arises from unit propagation, $c_i$ is not a sound RUP clause and we reject the proof. Performing this check for the entire sequence *C* is a means of verifying the result of *S*.

Propagating each clause in *C* through *F* can be expensive, and for large derivations we cannot keep the entirety of $F \cup C$ in memory. Therefore, a popular extension of proof formats allows the *deletion* of clauses [22]. Whenever *S* deletes a clause, it logs this deletion just like it logs learned clauses. This deletion can then be mirrored by the proof checker traversing the proof. A proof certificate now takes the shape ${\mathcal {C}} := \langle a_1, a_2, \ldots , a_{n'} \rangle$ where $a_i = (\textit{op}, c_i)$, $\textit{op} \in \{\textit{add}, \textit{delete}\}$, and $c_i$ is a clause.

The current standard format for proofs of unsatisfiability in sequential SAT solving is called DRAT [22], which allows for additions and deletions as outlined above. Each added clause must adhere to the so-called *RAT criterion* [22], a generalization of RUP that we do not detail. The more recent LRAT proof format [16] augments each clause addition with *hints*, or *dependencies*, that identify the clauses that were required to derive the current clause. This makes proof checking more efficient, and in fact the usual pipeline for trusted proof checking is first to use a fast unverified tool (e.g., [22]) to transform a DRAT proof into an LRAT proof, and then check the resulting LRAT proof with a formally verified proof checker [16, 29, 31, 47].

**Fig. 1:**
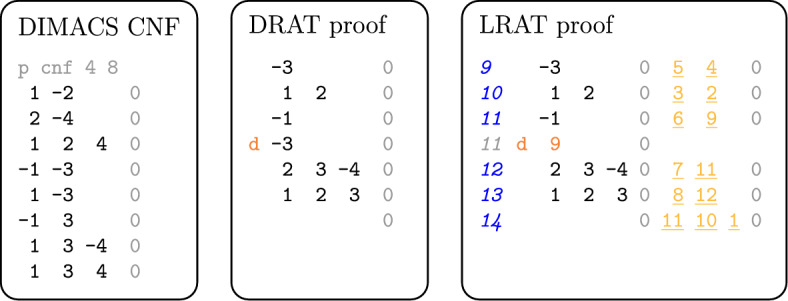
CNF formula and corresponding DRAT and LRAT proofs of its unsatisfiability. Headers and separators are set in light gray, deletions begin with “d”, leading clause IDs are italicized, and hints are underlined. Clause literals are always colored black

Figure 1 shows a formula (left) and its corresponding DRAT proof (center) and LRAT proof (right). Each proof line in the LRAT proof starts with a clause ID. The numbering starts with ID 9 because the eight clauses of the original formula are assigned the IDs 1 to 8. Each clause addition first lists the literals of the clause and then the clause’s dependencies in the form of clause IDs. Clause deletions only state the ID(s) of the clauses to delete, as in the later deletion of clause |9|. In our work, we exploit the hints of LRAT to determine dependencies among distributed solvers.

### Parallel and Distributed SAT Solving

The most common way to parallelize general-purpose SAT solving is to run a portfolio of sequential (mostly CDCL) solvers in parallel and to consider a problem solved as soon as one of the solvers finishes (c.f. [2–4, 17, 21, 32]). Given that the solvers are sufficiently diverse, portfolio solving is *effective* if all of the sequential solvers work independently but not *efficient* since only a single thread contributes to the final result. Scalability can be boosted significantly by having the solvers share information in the form of learned clauses [21]. This approach is taken by the distributed solver MallobSat [44, 45], which has dominated the cloud track of the International SAT competition since 2020 [15, 19, 41]. MallobSat relies on a communication-efficient aggregation strategy to collect the globally most useful distinct learned clauses and reliably to filter duplicates as well as previously-shared clauses [44]. This strategy aims to maximize the density and utility of the communicated data. As a matter of fact, MallobSat’s clause sharing was recently shown to be the main driver of its scalability, even if diversification is reduced to an absolute minimum, prompting its authors to use the term *clause-sharing solver* going forward [45].

Producing proofs of unsatisfiability is trivial for *pure* portfolio solvers if each of the employed solvers is able to output a proof itself. For clause-sharing portfolios, on the other hand, the derivation of the empty clause can depend on a clause produced by another solver, which may again depend on clauses from other solvers, and so on. The full chain of reasoning for a formula’s unsatisfiability can thus be a dense and interleaved network that features conflict clauses from all participating solvers.

Prior work on generating proofs from clause-sharing portfolio solvers is limited to shared-memory parallelism and cannot be generalized to distributed memory in any obvious manner. The recent shared-memory solver Gimsatul [7] is designed specifically to support outputting DRAT proofs; however, sequential checking of these proofs is “*most likely is too slow to be run in practice*” [7] and we are not aware of any notable parallel DRAT (or LRAT) checking approaches. In terms of generic clause-sharing portfolios, Heule et al. [27] attempted to generate proofs by having the solver threads emit proof lines concurrently into a single proof. Clause deletion statements can be added to the proof only after *all* solvers have reported deletion of the clause. Heule et al. obtained mixed results and for the most part were not able to arrive at proofs that are feasible to check, mostly due to the sheer size of the output and the large number of clauses that the checker is required to keep in memory.

## Basic Proof Production

Our goal is to produce checkable unsatisfiability proofs for problems solved by distributed clause-sharing SAT solvers. We propose to reuse the work done on proofs for sequential solvers by having each solver produce a partial proof containing the clauses it learned. These partial proofs are invalid in general because each sequential solver can rely on clauses shared by other solvers when learning new clauses. For example, when solver *A* derives a new clause, it might rely on clauses from solvers *B* and *C*, which in turn relied on clauses from solvers *D* and *E*, and so on. The justification of *A*’s clause derivation is thus spread across multiple partial proofs. We need to combine the partial proofs into a single valid proof in which the clauses are in *dependency order*, meaning that each clause can be derived from previous clauses.

In order to produce efficiently checkable proofs in a scalable manner, we address the following three challenges:

1. Provide metadata to identify which solver produced each learned clause.
2. Efficiently sort learned clauses in dependency order across all solvers.
3. Reduce proof sizes by removing unnecessary clauses.

Switching from DRAT to the LRAT proof format provides the mechanism to unlock all three challenges. First, we specialize the clause-numbering scheme used by LRAT in order to distinguish the clauses produced by each solver. Second, we use the dependency information from LRAT to construct a complete proof from the partial proofs produced by each solver. Finally, we determine which clauses are unnecessary (or used only for parts of the proof) to trim the proof where possible.

### Partial Proof Production

To combine the partial proofs into a complete proof, we modify the mechanism used to produce LRAT proofs in each of the individual sequential solvers. We assign to each clause an ID that is unique across solvers and identifies which solver originally derived it. The following mapping from clauses to IDs achieves uniqueness across solvers:

#### Definition 1

Let *o* be the number of clauses in the original formula and let *p* be the number of sequential solvers. Then the ID of the *k*-th derived clause ($k \ge 0$) of solver *i* is defined as $\textit{ID}^{i}_{k} = o + i + pk$.

Given $\textit{ID}^{i}_{k}$, we can easily determine the producing solver *i* using modular arithmetic.

We extend our clause sharing to send each clause together with its ID. A receiving solver stores the clause with its ID and uses the ID in proof hints when the clause is used locally, as it does with locally-derived clauses. Unlike locally-derived clauses, we add no derivation lines for incoming clauses to the local proof. Instead, these derivations will be added to the final proof when combining the partial proofs.

### Partial Proof Combination

Once the distributed solver reports unsatisfiability, we have *p* partial proofs. The derivations in these proofs can refer to clauses of other partial proofs, but they are locally in dependency order. We can thus combine the partial proofs without reordering their clauses beforehand. We can simply *interleave* their clauses so the resulting proof is also in dependency order, ignoring any deletions in the partial proofs.

Our *combination algorithm* traverses the partial proofs round-robin. At each step, we read and output the next clause *c* from the current partial proof as long as all dependencies of *c* have already been output. Checking whether a dependency *d* has already been output is simple: We determine which solver produced *d* (see Definition 1) and check if the next clause of the corresponding partial proof has an ID higher than *d*. Our algorithm terminates when it emits the empty clause.

**Fig. 2:**
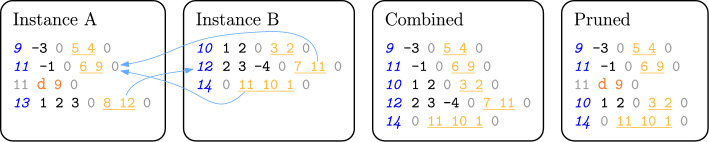
Partial proofs and combined and pruned proof, colored as in Fig. 1. Arrows indicate remote dependencies across the partial proofs

Suppose that two clause-sharing solver instances, A and B, found the formula from Fig. 1 to be unsatisfiable and emitted two partial proofs as shown in Fig. 2. Starting with A, we can emit clause 9 (only depending on original clauses) and 11 (depending on original clauses and clause 9). We cannot emit clause 13 since it depends on clause 12 from B. Proceeding with B, we can now emit the remaining clauses 10, 12, and 14. Since clause 14 is the empty clause, we finish with the complete proof shown in Fig. 2 (middle right). Note that clause 13 was not added to the combined proof as it was not required to satisfy any dependencies of the empty clause.

### Proof Pruning

The combined proof our procedure produces is valid but not efficiently checkable because (1) it can contain superfluous clauses and (2) it does not contain deletion lines, meaning that a proof checker must maintain *all* learned clauses in memory throughout the checking process. To reduce size and improve checking performance, we *prune* our combined proof to contain only necessary clauses, and we add deletion statements for clauses as soon as they are not needed anymore.

Our *pruning algorithm* walks the combined proof *in reverse* to find all transitive dependencies of the empty clause, similar to backward checking of DRAT proofs [26]. We maintain a set *R* of clause IDs *required* in the proof, initialized to the ID of the empty clause alone. We then read all clauses in reverse order, including the empty clause. Clauses that are not required are ignored. When encountering a clause derivation whose ID is in *R*, we check for each of its dependencies whether this is the first time (from the proof’s end) we see this dependency. In such cases, we can emit a deletion line for the dependency since it is the last time the clause is used in the proof. After checking all its dependencies, we output the required clause derivation and add its dependencies (except for original clauses) to *R*. The final output of the algorithm is a proof in reversed order, where each clause is required for some derivation and deleted as soon as it is no longer required. Reversing this output line by line yields a sound and compact proof.

Consider the combined proof in Fig. 2. Working backward from clause 14, with clause IDs 11 and 10 added to *R*, we determine that clause 12 is *not* required, so it is ignored. Additionally, prior to clause 11, clause 9 is not in *R*, so it can be deleted after the derivation of clause 11. As such, we arrive at the pruned proof in Fig. 2.

On realistic proofs, we show in Sect. 6 that pruning can sometimes reduce the proof size by several orders of magnitude.

## Distributed Proof Production

The proof production as described above is sequential and may process huge amounts of data, all of which needs to be accessible from the machine that executes the procedure. In addition, maintaining the required clause IDs during the procedure can require a prohibitive amount of memory for large proofs. In the following, we propose an efficient distributed approach to proof production that addresses these scalability issues by exploiting some key properties of periodic all-to-all clause sharing.

### Overview

Our sequential algorithm first combines all partial proofs into a single proof and then prunes unneeded proof lines. In contrast, our distributed algorithm first prunes all partial proofs in parallel and only then merges the required lines into one file.

**Fig. 3:**
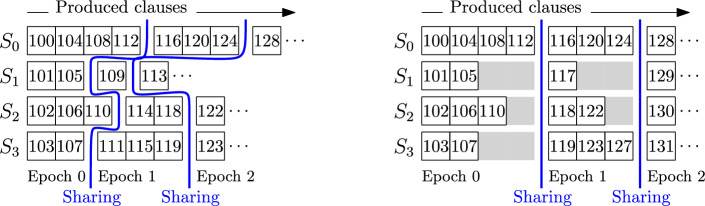
Four solvers work on a formula with 99 original clauses, produce new clauses (depicted by their ID) and share clauses periodically, without (left) and with (right) clause ID alignment

As our distributed execution environment, let us assume a two-level hierarchy where we have *m* *processes* which run $c \ge 1$ solver threads each, amounting to a total of $p = mc$ solvers. Furthermore, we assume that clauses are shared in an *all-to-all* fashion (i.e., every solver may receive clauses from every other solver) in periodic intervals. Both of these assumptions hold for the popular distributed systems HordeSat [3] and MallobSat [44]. We refer to the intervals between subsequent sharing operations as *epochs*. Consider Fig. 3 (left): Clause 118 was produced by $S_2$ in epoch 1. Its derivation may depend on local clause 114 and on any of the 11 clauses produced in epoch 0, but it cannot depend on, e.g., clause 109 or 111 since these clauses have been produced after the last clause sharing. More generally, a clause *c* produced by solver *i* during epoch *e* can only depend on (1) earlier clauses by solver *i* produced during epoch *e* or earlier, and (2) clauses by solvers $j \ne i$ produced *before* epoch *e*.

Using this knowledge, we can *rewind* the solving procedure. Each process reads its partial proofs in reverse, outputs each line that adds a required clause, and adds the line’s dependencies to the required clauses. Required *remote* clauses produced in epoch *e* are transferred to their origin before any process begins to read proof lines from epoch *e*. As such, whenever a process reads a proof line, it knows if the clause is required. We later explain how the outputs of all processes can be merged.

### Clause ID Alignment

To synchronize the reading and redistribution of clause IDs in our distributed pruning, we need a way to decide from which epoch a remote clause ID originates. However, solvers generally produce clauses with different speeds, so the IDs by different solvers will likely be in dissimilar ranges within the same epoch over time. For instance, in Fig. 3 (left) solver $$S_3$$ has no way of knowing from which epoch clause 118 originates. To solve this issue, we propose to align all produced clause IDs after each sharing. During solving, we add a certain offset $$\delta _i^e$$ to each ID produced by solver *i* in epoch *e*. As such, we can associate each epoch *e* with a global interval $$[ A_e, A_{e+1} )$$ that contains all clause IDs produced in that epoch. In Fig. 3 (right), $$A_0 = 0$$, $$A_1 = 116$$, and $$A_2 = 128$$. Clause 118 on the left has been aligned to 122 on the right ($$\delta _2^1 = 4$$) and due to $$A_1 \le 122 < A_2$$ *all* solvers know that this clause originates from epoch 1.

Initially, $\delta _i^0 := 0$ for all *i*. Let $I_i^e$ be the first original (unaligned) ID produced by solver *i* in epoch *e*. With the sharing that initiates epoch $e>0$, we want to define the common start of epoch *e*, $A_e$, to be larger than all aligned clause IDs from epoch $e-1$ but no larger than any aligned clause ID from epoch *e*. We align each solver’s first ID from epoch *e* via the prior offset: $I_{i}^e + \delta _{i}^{e-1}$. We then normalize each such ID by subtracting the solver’s individual offset *i*. Since two subsequent unaligned clause IDs always differ by *p* (the total number of solvers) and since $i<p$, we know that $I_{i}^e + \delta _{i}^{e-1} - i$ is larger than the last ID solver *i* produced in epoch $e-1$. We thus compute $A_e$ as the maximum of these values: $A_e := \max _{i}\{ I_{i}^e + \delta _{i}^{e-1} - i\}$. Next, we want to compute new offsets $\delta _i^e$ in such a way that the first aligned clause ID of solver *i* in epoch *e*, $I_i^e + \delta _i^e$, is equal to $A_e + i$. Consequently, we set $\delta _i^e := A_e + i - I_i^e$.

If we export a clause produced in epoch *e* by solver *i*, we add $\delta _i^e$ to its ID, and if we import shared clauses to *i*, we filter any clauses produced by *i* itself. Note that we do not modify the solvers’ internal ID counters nor the proofs they output, and there is no need to block or synchronize the solver threads at any time. Later, when reading the partial proof of solver *i* at epoch *e*, we need to add $\delta _i^e$ to each ID originating from solver *i*. All remote clause IDs in the partial proofs are already aligned.

### Rewind Algorithm

Assume that solver $u \in \{1,\ldots ,p\}$ has derived the empty clause in epoch ${\hat{e}}$. Each process has a *frontier* $R_i$ for each process-local solver *i*. Each $R_i$ features the required clauses produced by *i*. In addition, each process has a *backlog* *B* of remote required clauses. *B* and $R_i$ are maximum-first priority queues of clause IDs. Initially, $R_u$ contains the ID of the empty clause while all other frontiers and backlogs are empty. Iteration $x \ge 0$ of our algorithm processes epoch ${\hat{e}}-x$ and features two stages:

1. *Processing:* Each process continues to read its partial proofs in reverse order from the final derived clause of the current epoch. If a line from solver *i* is read whose clause ID is at the top of $R_i$, then the ID is removed from $R_i$, the line is output, and each clause ID hint *h* in the line is treated as follows:
  *h* is inserted in $R_{j}$ if local solver *j* (possibly $j=i$) produced *h*.
  *h* is inserted in *B* if a remote solver produced *h*.
  *h* is ignored if *h* is an ID of an original clause of the problem.
  Reading stops as soon as a line’s ID precedes epoch $e = {\hat{e}}-x$. Each $R_i$ as well as *B* now only contain clauses produced *before* *e*.
2. *Task redistribution:* Each process extracts all clause IDs from *B* that were produced during ${\hat{e}}-x-1$. These clause IDs are aggregated among all processes. In our concrete implementation, we reuse MallobSat’s compact clause exchange operation [45], adjusted to aggregate clause IDs instead of clauses. This also allows us to eliminate duplicates among the redistributed IDs. Each process then traverses the aggregated clause IDs, and each clause produced by a local solver *i* is added to $R_i$.

Our algorithm stops in iteration ${\hat{e}}$ after the processing stage, at which point all frontiers and backlogs are empty and all relevant proof lines have been output. The result is one partial proof per solver, with each partial proof containing the lines output by the corresponding solver.

### Correctness

We now establish the correctness of our proof production. First, we show that our clause ID alignment works as intended:

#### Lemma 1

The alignment of clause IDs as described above results in a sequence $A_0, A_1, A_2, \ldots , A_{{\hat{e}}}$ such that for any clause with unaligned ID *j* produced by the *i*-th solver, $A_e \le j+\delta _i^e < A_{e+1}$ holds if and only if *j* was produced in epoch *e*.

#### Proof

We perform induction over epoch *e* in which a clause was produced.

For $e=0$, we set $A_0 = 0$. The first sharing defines $A_1 = \max _{i}\{ I_{i}^1 + \delta _{i}^{0} - i\} = \max _{i}\{ I_{i}^1 - i\}$.

$I_i^1$ is the first clause ID the *i*-th solver produced in epoch 1 and *i* is smaller than the difference *p* between two of its subsequent clause IDs. Therefore, $I_i^1 - i$ is larger than any ID it produced in epoch 0. Consequently, $A_1$ is larger than any ID produced in epoch 0 by *any* solver. It follows that a clause with ID *j* was produced in epoch 0 if and only if $A_0 = 0 \le j < A_1$.

Assuming that the lemma holds for clauses produced in epochs $0,\ldots ,e$, we show that the lemma also holds for clauses produced in epoch $e+1$.

Due to induction, a clause from the *i*-th solver with unaligned ID *j* was produced in epoch *e* if and only if $A_e \le j+\delta _i^{e} < A_{e+1}$. We need to show that a clause with unaligned ID *j* was produced in epoch $e+1$ if and only if $A_{e+1} \le j+\delta _i^{e+1} < A_{e+2}$.

The induction prerequisite enforces that $A_{e+1}$ exactly separates the aligned clause IDs produced in epoch *e* from the aligned clause IDs produced in later epochs. Therefore, $A_{e+1} \le j+\delta _i^{e+1}$ if and only if *j* was produced in epoch $e+1$ *or later*.

Concerning the upper bound, our procedure defines $A_{e+2} = \max _{i}\{ I_i^{e+2} + \delta _i^{e+1} - i \} = \max _{i}\{ I_i^{e+2} + (A_{e+1}+i) - I_i^{e+1} - i \} = A_{e+1} + \max _{i}\{ I_i^{e+2} - I_i^{e+1} \}$. Since $\delta _j^{e+1} = A_{e+1} + i - I_i^{e+1}$, it follows that $j+\delta _i^{e+1} < A_{e+2}$ holds if and only if $j + i - I_i^{e+1} < \max _{i}\{ I_i^{e+2} - I_i^{e+1} \}$, which is equivalent to (A) $j < I_i^{e+1} + \max _{i}\{ I_i^{e+2} - I_i^{e+1} \} - i$. Since the first clause ID produced by the *i*-th solver in epoch $e+2$ is $I_i^{e+2} \ge I_i^{e+1} + \max _{i}\{ I_i^{e+2} - I_i^{e+1} \} - i$, (A) holds if and only if *j* was produced *before* epoch $e+2$. $\square$

Next, we need to formally define a partial proof for an individual solver thread.

#### Definition 2

Let *S* be a sequential solver that runs within a distributed clause-sharing solver. A *partial proof* for CNF formula *F* is a sequence ${\mathcal {P}} = \langle l_1, \ldots , l_n \rangle$ of LRAT proof lines output by *S* without any clause deletions, where for each line $l_i = (j, c, D)$ with ID *j*, clause *c*, and dependencies *D*, (i) and (ii) hold:

(i) Each dependency $d \in D$ references either (a) an original clause in *F* or (b) a clause derived in an earlier line $l_j$ ($j < i$) or (c) a clause from another partial proof for *F*. There must not be any cyclic dependencies.
(ii) $l_i$ constitutes a valid LRAT derivation of *c* if given the referenced dependencies.

The following theorem states the correctness of our proof production under the assumption that the individual solvers output valid partial proofs.

#### Theorem 1

Let ${\mathcal {P}}_1, \ldots , {\mathcal {P}}_m$ be the partial proofs for an unsatisfiable CNF formula *F* of a completed run of a distributed solver that performs all-to-all clause sharing with clause ID alignment as outlined above. Let $O := \langle O_1, \ldots , O_m \rangle$ be the proof line output of each solver thread from our rewind procedure, and let ${\tilde{O}}$ be a flat sequence of all proof lines in *O* sorted by ID in ascending order. Then ${\tilde{O}}$ constitutes a sound LRAT proof for *F*.

#### Proof

First, we state that ${\tilde{O}}$ contains the empty clause due to construction: Since *F* is unsatisfiable and the distributed solver’s run completed, the empty clause has been found by at least one solver and is consequently output by some solver at the beginning of the rewind procedure.

Next, we show for any line $l = (j, c, D) \in {\tilde{O}}$ that a linear pass through ${\tilde{O}}$ establishes all dependencies $d \in D$ before *l* itself is reached. Since $l \in {\tilde{O}}$, there is a solver *i* whose partial proof ${\mathcal {P}}_i$ contains *l* in epoch *e* and where *j* is considered required such that *l* is output. We distinguish three cases:

- If *d* references an original clause in *F*, the dependency is trivially established.
- If *d* references an earlier clause derived in ${\mathcal {P}}_i$, then dependency *d* is inserted in $R_i$ as *l* is read from ${\mathcal {P}}_i$. We know that $d < j$: Each solver assigns clause IDs in a strictly monotonic manner and the alignment of clause IDs preserves this property. Since the derivation of *d* is contained in ${\mathcal {P}}_i$ and since the IDs in ${\mathcal {P}}_i$ are processed in decreasing order, the line deriving *d* is read from ${\mathcal {P}}_i$ at some later point in time. At this point, *d* must be at the top of $R_i$ for the following arguments. IDs extracted from $R_i$ are monotonically decreasing because $R_i$ functions as a maximum-first priority queue and because each ID inserted in $R_i$ is necessarily smaller than the last ID extracted from $R_i$. If a higher ID $d' > d$ is at the top of $R_i$, then the required dependency $d'$ was not matched with any former line in ${\mathcal {P}}_i$ and, due to the processing order of IDs in ${\mathcal {P}}_i$, is not matched with any later line either. As our procedure ensures that $R_i$ only contains IDs produced by solver *i*, this constitutes a contradiction to ${\mathcal {P}}_i$ being a valid partial proof. If a lower ID is at the top of $R_i$ or if $R_i$ is empty, then *d* was removed from $R_i$ earlier, which means that *d* can be matched with several lines from ${\mathcal {P}}_i$—a contradiction to the uniqueness of derived clause IDs in ${\mathcal {P}}_i$. Since *d* is at the top of $R_i$ as its derivation is read from ${\mathcal {P}}_i$, *d* is considered required and thus output. Due to $d<j$, dependency *d* is in fact established before *l* is reached.
- In the third case, *d* references a clause $c'$ from another solver’s partial proof ${\mathcal {P}}'$. Due to the structure of clause sharing, any such remote clause $c'$ originates from a strictly smaller epoch $e'$ than the epoch *e* from which *l* itself originates. Our clause ID alignment (Lemma 1) hence ensures that the ID *d* of $c'$ is strictly smaller than *j* and that *d* would be featured in ${\tilde{O}}$ earlier than *l*. It remains to be shown that ${\tilde{O}}$ does contain *d*. Since *l* is output, the remote dependency *d* is inserted either in backlog $B_i$ (if *d* originates from a different process) or directly in the producing solver’s frontier $R'$. In the former case, before epoch $e'$ is processed, *d* is extracted from $B_i$, redistributed to the producing solver, and *then* inserted in that solver’s frontier $R'$. During the processing of epoch $e'$, the derivation of *d* is read from ${\mathcal {P}}'$. At this point in time, the ID *d* must be at the top of $R'$ for exactly the same arguments as in case (b) for $R_i$. Since *d* is at the top of $R'$, *d* is considered required and therefore output as well.

All in all, ${\tilde{O}}$ is a sequence of valid LRAT derivations in dependency order that eventually features the empty clause. Therefore, ${\tilde{O}}$ constitutes a sound LRAT proof of unsatisfiability for *F*. $\square$

To complement Theorem 1, we can also argue that any proof line $l \in {\tilde{O}}$ is necessarily a transitive requirement of the empty clause and that, therefore, ${\tilde{O}}$ is minimal in the sense that all lines in ${\tilde{O}}$ are in some way required for the proof at hand. There can still be ways to achieve smaller proofs for *F*. For instance, the same clause *c* may be featured multiple times with different IDs in ${\tilde{O}}$ or there may be an entirely different, shorter chain of reasoning leading to the empty clause.

### Analysis

In terms of total work performed, all partial proofs are read completely. For each required clause we may perform an insertion into some *B*, a deletion from said *B*, an insertion into some $R_i$, and a deletion from said $R_i$. If $V_{in}$ is the combined size of all partial proofs, $V_{out}$ is the size of the output proof, and we assume logarithmic work for each insertion and deletion, then the work for these operations is in ${\mathcal {O}}(V_{in} + V_{out} \log V_{out})$. In addition, due to the redistribution of clause IDs we have ${\hat{e}}$ iterations of communication whose overall cost is bounded by the communication done during solving. In fact, since only a subset of shared clauses is required and we only share 64 bits per clause, we expect strictly less communication than during solving. In addition, $A_e$ must be computed for each epoch *e* during solving, each of which requires the aggregation and broadcast of ${\mathcal {O}}(1)$ data. For the case of MallobSat, this computation can be integrated into the all-reduction of the clause filtering bitset [45] and is therefore fully negligible.

In terms of memory usage, the size of each *B* and each $R_i$ can be proportional to the combined size of all required lines of the according partial proofs. This memory requirement may become problematic for large-scale runs. We thus suggest to employ external-memory priority queues (e.g., [38]) which keep most of their data on disk.

### Merging Step

For each partial proof processed during the pruning step, we have a stream of proof lines sorted in reverse chronological order, i.e., starting with the highest clause ID. The remaining task is to merge all these lines into a single, sorted proof file.

**Fig. 4:**
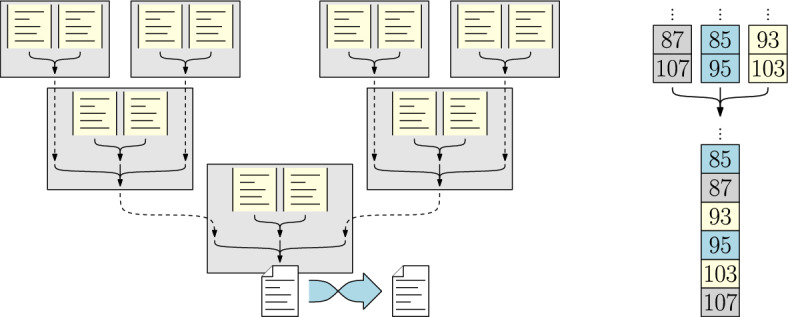
Left: Proof merging with seven processes and 14 solvers. Each box represents a process with two local proof sources. Dashed arrows denote communication. Right: Example of merging three streams of LRAT lines into a single stream. Each number *i* represents an LRAT line describing a clause of ID *i*

We arrange all processes in a *k*-ary tree as shown in Fig. 4 (left) for $k=2$. At each node of this tree, we can easily merge a number of sorted input streams into a single sorted output stream by repeatedly outputting the line with the highest ID among all inputs (Fig. 4 right). This way, we can hierarchically merge all streams along the tree. At the tree’s root, the output stream is directed into a file. This is a sequential I/O task that limits the speed of merging. Finally, since the produced file is in reverse order, a buffered operation reverses the file’s content.

In general, there may be more scalable ways to sort the available proof information (e.g., [30]), especially if we allow several processes to output slices of the final sorted proof in parallel. However, our algorithm assumes that (a) we require a single proof file on a single process and (b) the proof volume is so large that we need to stream it from disk memory. Moreover, since other steps in our pipeline (postprocessing and proof checking, see Sect. 6.1) process the final proof sequentially, our merging approach does not constitute a bottleneck in and of itself.

A final challenge is to add clause deletion statements to the final proof. Before a line is written to the combined proof file, we can scan its hints and output a deletion line for each hint we did not encounter before (see Sect. 3.3). However, implementing this in an exact manner requires maintaining a set of clause IDs which scales with the final proof size. Since clause deletions are inserted for efficiency and do not affect soundness, we can use an approximate membership query (AMQ) structure with fixed size and a small false positive rate, e.g., a Bloom filter [11].

## Base Implementation

We now outline our implementation as in our original conference paper [35].

We employ a solver portfolio based on the sequential SAT solver CaDiCaL [8]. We modified CaDiCaL to output LRAT proof lines and to assign clause IDs as described in Sect. 3.1. To ensure sound LRAT proof logging, we need to turn off some features of CaDiCaL, such as bounded variable elimination, hyper-ternary resolution, and vivification. Similarly, MallobSat’s original portfolio of CaDiCaL configurations features several options that are incompatible with our CaDiCaL as of yet. We thus created a smaller portfolio of “safe” configurations that include shuffling variable priorities, adjusted restart intervals, and disabled inprocessing. We also use different random seeds and sparse random variable phases.

Throughout this work, we use the distributed clause-sharing solver MallobSat [45] (see Sect. 2.3) both as a basis for our approach and also as a primary competitor for experiments involving non-proof-producing approaches. We modified MallobSat [40] to associate each clause with a 64-bit clause ID. For consistent bookkeeping of sharing epochs, we defer clause sharing until all processes have fully initialized their solvers. While several solvers may derive the empty clause simultaneously , only one of them is chosen as the “winner” whose empty clause will be traced. The distributed proof production features communication epochs similar to MallobSat’s clause sharing. To keep memory requirements of our proof assembly manageable even for huge proofs, we implement the clause ID priority queues $R_i$ and *B* with a simple semi-external data structure based on an in-memory priority queue *Q* for the current epoch and one external-memory stack $E_e$ for each epoch *e* still to be processed. Upon reaching a new epoch *e*, all clause IDs from *e* are read from $E_e$ and inserted into *Q* to allow for efficient polling and insertion.

To merge the pruned partial proofs, we use point-to-point messages to query and send buffers of proof lines between processes. We perform pruning and merging simultaneously to avoid writing the pruned partial proofs to disk. We use a fixed-size Bloom filter to add deletion lines to the final proof.

## Base Evaluation

In this section, we present an evaluation of our proof production approaches with our base implementation. We provide all software and experimental data online.^2^

### Experimental Setup

Supporting proofs introduces several kinds of performance overhead for clause-sharing portfolios in terms of solving, proof reconstruction, and proof checking. We wish to examine how well our proof-producing solver performs against (1) state-of-the-art (massively) parallel solvers that do not produce proofs, (2) previous approaches to proof-producing parallel solvers, and (3) state-of-the-art sequential solving with and without proof production. We analyze the overhead introduced by each phase of the process, and we discuss how and where future efforts might improve performance.

We use the following pipeline for our proof-producing solvers: First, the input formula is preprocessed via exhaustive unit propagation—a necessity due to a technical limitation of our LRAT-producing modification of CaDiCaL. Second, we execute our proof-producing variant of MallobSat on the preprocessed formula. Third, we prune and combine all partial proofs, using either our sequential proof production or our distributed proof production. Fourth, we merge the preprocessor’s proof and our produced proof, compressing all clause IDs into a compact domain. Fifth and finally, we run lrat-check^3^ to check the final proof. Only steps two and three of this pipeline are parallelized (step three depending on the particular experiment). We refer to the first two steps as *solving*, the third step as *assembly*, the fourth step as *postprocessing*, and the fifth step as *checking*.

To analyze solving performance, we compare our parallel (MallobSatP64) and cloud (MallobSatP1600) solvers with proof production to several other solvers. First, we include the winners of the ISC 2022 cloud track (MallobSat1600-KCLG [40], see Sect. 2.3, using Kissat, CaDiCaL, Lingeling, Glucose), parallel track (ParkissatRS[50], using Kissat), and sequential track (KissatMABHyWalk [51]), as well as the recent shared-memory parallel solver Gimsatul^4^ which also supports proof production. In addition, we reconfigured MallobSat1600-KCLG to use only CaDiCaL (its original version, i.e., without LRAT capabilities) with the restricted configuration options used by MallobSatP1600 and MallobSatP64. We run this solver on a parallel (MallobSat64-C) and cloud (MallobSat1600-C) scale.

Since prior work on proof production for clause-sharing portfolios [27] is no longer competitive in terms of solving time, we only compare proof-checking times. Specifically, we measure the overhead of checking un-pruned DRAT proofs as produced by the earlier approach [27]. As such, we can get a picture of the performance of the earlier approach if it was realized with today’s solving techniques. We generate un-pruned DRAT proofs from the original (un-pruned) LRAT proof by stripping out dependency information and adding delete lines for the last use of each clause.

We ran our experiments in Amazon Web Services (AWS) infrastructure. Specifically, following the ISC setup, each cloud solver runs on 100 m6i.4xlarge EC2 instances (16 hardware threads, 64 GB RAM), each parallel solver runs on a single m6i.16xlarge EC2 instance (64 hardware threads, 256 GB RAM), and the sequential KissatMABHyWalk runs on a single m6i.4xlarge EC2 instance. We use all 400 benchmark instances from ISC 2022. We set the timeout for the solving step to 1000 s and the timeout for all subsequent steps put together to 4000 s.

### Results

**Fig. 5:**
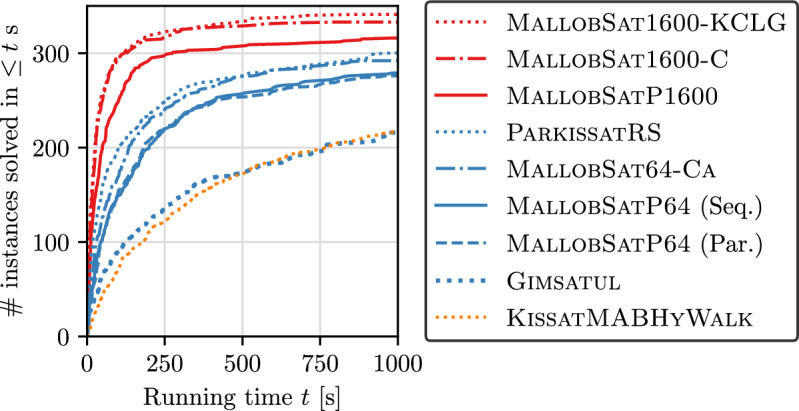
Solving times of considered solvers (higher is better). MallobSatP1600, MallobSatP64, and KissatMABHyWalk output proof information during solving

First we examine the performance overhead of changing portfolios to enable proof generation (see Sect. 5) regarding solving times *only*. Fig. 5 and Table 1 show this data. Our CaDiCaL portfolio MallobSat64-C drastically outperforms KissatMABHyWalk as well as Gimsatul and is almost on par with ParkissatRS. Similarly, MallobSat1600-C solves eight instances less than MallobSat1600-KCLG but performs almost equally well otherwise. In both cases, we have constructed solvers that are almost on par with the state of the art.

**Table 1:** Performance of (S)equential, (P)arallel, and (C)loud solvers in terms of solved instances (#), also divided in satisfiable and unsatisfiable instances, and PAR-2 score, i.e., the arithmetic mean running time where timeouts are counted as solved in twice the time limit

Type	Solver	#	# SAT	# UNSAT	PAR-2
S	KissatMABHyWalk	218	118	100	1065.7
P	ParkissatRS	300	155	145	603.0
	Gimsatul	216	119	97	1058.0
	MallobSat64-C	292	145	147	641.6
	MallobSatP64 (Seq.)	279	140	139	719.8
	MallobSatP64 (Par.)	276	141	135	731.4
C	MallobSat1600-KCLG	341	165	176	344.8
	MallobSat1600-C	333	163	170	378.0
	MallobSatP1600	316	159	157	480.5

For our proof-producing solvers MallobSatP64 and MallobSatP1600, we noticed a more pronounced decline in solving performance. Note that a later version of our system overcomes most of the underlying technical issues, resulting in better performance (Sect. 7). That being said, the proof-producing solvers discussed at this point do already outperform all of the solvers at a lower scale.

Next, we examine statistics on proof reconstruction and checking, showing results in Table 2. Since we want to investigate our approaches’ overhead compared to pure solving, we measure running times as a multiple of the solving time. Table 3 shows results in terms of absolute running times. The suffix “Seq.” denotes MallobSatP64 with sequential proof production, “Par.” denotes MallobSatP64 with distributed proof production run on a single machine, and “Cld.” denotes MallobSatP1600 with distributed proof production.

**Table 2:** Statistics on proof production and checking, considering a prior DRAT-based approach [27] (64 threads), our approach at 64 threads with sequential (Seq.) and parallel (Par.) proof production, and our approach at the cloud scale (Cld., 1600 threads)

Property	#	Min	p10	Med	Mean	p90	Max
DRAT check	81	0.512	1.725	7.442	10.370	67.065	169.869
Seq. assembly	139	0.019	0.305	1.376	1.387	5.747	13.289
Seq. postprocessing	139	0.001	0.012	0.131	0.112	0.790	2.218
Seq. checking	139	0.007	0.043	0.572	0.469	3.970	10.980
Seq. asm+post+chk	139	0.037	0.412	2.110	2.129	10.834	26.487
Par. assembly	135	0.059	0.080	0.365	0.408	2.227	7.475
Par. postprocessing	135	0.001	0.016	0.156	0.128	0.861	2.300
Par. checking	135	0.007	0.042	0.622	0.471	3.540	11.645
Par. asm+post+chk	135	0.067	0.167	1.097	1.062	6.611	21.420
Cld. assembly	157	0.121	0.194	1.680	1.204	5.348	43.853
Cld. postprocessing	157	0.003	0.051	0.744	0.634	4.744	35.667
Cld. checking	157	0.032	0.215	3.391	2.499	21.908	135.737
Cld. asm+post+chk	157	0.162	0.579	5.174	4.819	31.968	215.257
DRAT proof size (GB)	139	0.012	0.366	1.236	3.246	8.395	29.308
Seq. proof size (GB)	139	0.016	0.223	2.379	5.384	16.082	46.986
Par. proof size (GB)	135	0.006	0.173	2.034	5.345	13.164	57.739
Cld. proof size (GB)	157	0.016	0.269	4.595	11.138	34.457	92.276
Cld. pruning factor	157	2.080	5.312	16.472	28.319	299.858	8415.070

All properties except for file sizes and pruning factor are given as a multiple of the solving time. We list minima, maxima, medians, means, and the 10th and 90th percentiles—using the arithmetic mean for proof sizes and the geometric mean for all ratios

**Table 3:** Statistics on proof production and checking given *in seconds*

	#	Min	p10	Med	Mean	p90	Max
DRAT check	81	24.564	161.947	636.053	1025.771	2675.848	3399.476
Seq. assembly	139	6.141	37.998	158.011	277.023	747.614	1571.190
Seq. postprocessing	139	0.120	1.695	13.776	31.376	87.583	231.958
Seq. checking	139	0.716	7.627	60.587	140.934	368.082	1200.319
Seq. asm+post+chk	139	7.924	62.542	242.040	449.334	1208.350	2831.480
Par. assembly	135	2.196	10.763	41.781	96.167	231.383	1054.070
Par. postprocessing	135	0.202	1.552	16.708	34.587	82.215	338.245
Par. checking	135	0.867	5.157	59.240	148.206	377.040	1469.763
Par. asm+post+chk	135	3.406	18.492	113.739	278.960	697.353	2862.080
Cld. assembly	157	1.474	11.008	61.019	108.122	277.119	848.708
Cld. postprocessing	157	0.249	2.944	31.703	87.176	266.439	690.279
Cld. checking	157	1.141	9.564	130.755	347.430	1006.636	2626.983
Cld. asm+post+chk	157	3.626	36.400	217.736	542.728	1526.270	4165.970

DRAT checking succeeded in 81 out of 139 cases and timed out in 58 cases. For the successful cases, DRAT checking took 10.4$\times$ the solving time^5^ whereas our sequential assembly, postprocessing and checking *combined* succeeded in 139 cases and only took 2.1$\times$ the solving time. This result confirms that our approach successfully overcomes the major scalability problems of earlier work [27]. In terms of uncompressed proof sizes, our LRAT proofs can be about twice as large as the DRAT proofs, which seems more than acceptable considering the dramatic difference in performance. Given that DRAT-based checking was ineffective at the scale of parallel solvers, we decided to omit it in our distributed experiments that feature even larger proofs.

**Fig. 6:**
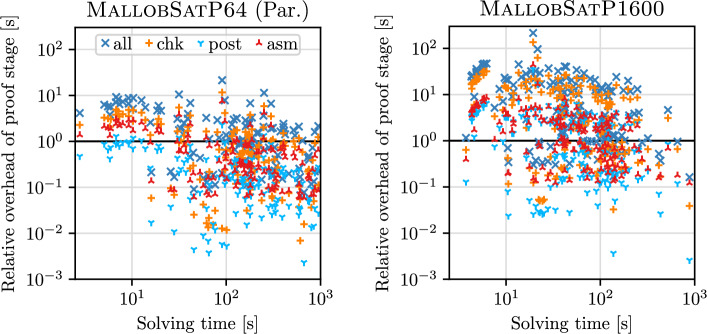
Overhead of proof-related stages (assembly, postprocessing, checking, and overall) relative to solving time, for MallobSatP64 with parallel proof production (left) and for MallobSatP1600 (right). Note the logarithmic scaling

The *parallel* proof production of MallobSatP64 reduces proof assembly times from 1.4$\times$ down to 0.4$\times$ the solving time, which also significantly reduces the overall overhead of proof production and checking (2.13$\times$ down to 1.06$\times$ the solving time). Figure 6 (left) illustrates these relative overheads (*y* direction, as multiples of solving time) in relation to the actual solving time (*x* direction).

The results for MallobSatP1600 demonstrate that our proof assembly is feasible, still taking only around 1.2$\times$ the solving time on average. In contrast, the sequential stages of postprocessing and checking do not scale and therefore become more noticeable relative to the solving time (see Fig. 6 right). The proofs produced are about twice as large as for MallobSatP64. Considering that the proofs originate from 25 times as many solvers, this increase in size is quite modest, which is partly due to our proof pruning. We captured the *pruning factor*—the number of clauses in all partial proofs divided by the number of clauses in the combined proof—for each instance. Our pruning reduces the derived clauses by a mean factor of 28.3 (median 16.4) and by a factor of 300 or more for 10% of all instances. This underlines that our pruning is a crucial technique feasibly to combine and check proofs. We also managed to produce and check a proof of unsatisfiability for a formula whose unsatisfiability has not been verified before to our knowledge (PancakeVsInsertSort_8_7.cnf).

To compare our approaches with the state of the art in sequential solving, we analyzed drat-trim checking times of KissatMABHyWalk (Table 4), kindly provided by the competition organizers, and arrived at a mean overhead of 1.2$\times$ its own solving time. Using this data, we computed the speedups of our parallel trusted approaches over KissatMABHyWalk—once where both the sequential and the parallel approach perform solving only and once where both approaches perform solving, proof production, and checking. To compute speedups, note that we use a conservative but clean approach where we only consider instances which both the sequential and the parallel solver were able to solve [45]. Also note that the sequential solvers in the ISC are executed on different hardware than the parallel solvers; as such, these speedup measures are not fully reliable and only meant to give a rough impression.

**Table 4:** DRAT-trim proof checking overhead of KissatMABHyWalk as recorded in the SAT Competition 2022, in terms of multiples of its solving time (“Ratio”) and in terms of absolute running times (“Time”)

	#	Min	p10	Med	(g/a)mean	p90	Max
Ratio	146	0.137	0.269	1.109	1.208	6.394	66.494
Time	146	8.978	59.325	576.400	2675.813	5246.770	(Timeout)

**Table 5:** Speedups of our approach over KissatMABHyWalk when only considering their solving times (top) and when considering their entire trusted solving and checking pipelines (bottom)

		#	Min	p10	Med	gmean	p90	Max	Total
slv only	Seq. (64$\times$)	263	0.028	0.742	3.869	3.806	23.224	122.854	4.429
	Par. (64$\times$)	260	0.051	0.865	3.786	3.831	21.147	901.653	4.599
	Cld. (1600$\times$)	283	0.100	2.179	10.887	11.253	64.624	1235.170	13.336
slv+chk	Seq. (64$\times$)	263	0.028	0.572	3.275	3.234	21.665	127.707	5.385
	Par. (64$\times$)	260	0.051	0.866	4.120	3.851	21.217	901.654	6.472
	Cld. (1600$\times$)	283	0.101	1.093	6.362	6.818	60.380	1235.170	7.226

Table 5 shows these speedups. The mean speedup in terms of pure solving times is about 4 with 64 solvers and about 11 with 1600 solvers—still comparable to the speedups which have earlier been reported by MallobSat’s precursor HordeSat (with no proof production capabilities) at similar scales [3]. In terms of the full trusted solving pipeline, our parallel proof production with 64 solvers actually achieves slightly larger speedups than if we consider only pure solving times—indicating that our LRAT-based proof production and checking pipeline is highly efficient and practical when compared to a sequential DRAT-based proof pipeline. The speedup at a distributed scale, by constrast, drops by roughly 40% when also considering the production and checking of proofs. As analyzed above, this is in large part due to the sequential and therefore non-scalable postprocessing and checking steps in our pipeline. While pre- and post-processing is a technical necessity in this setup, large portions of it can be eliminated with further engineering, as we outline in the following section. All in all, while the reported speedups of our base setup are still considerably below speedups achieved without proof production due to different kinds of overhead, they have served as encouraging results towards efficient trusted general-purpose SAT solving in distributed environments.

## Follow-Up

Since our original implementation (Sect. 5) and evaluation (Sect. 6), which remain an important point of reference, we have integrated further improvements in our system that we outline and assess in the following.^6^

### Improvements

Motivated by our original publication, Pollitt et al. [37] recently presented a new version of CaDiCaL supporting full LRAT proof output. Updating MallobSat’s CaDiCaL backend accordingly improves general solver performance, allows all of CaDiCaL’s configuration options, and, most importantly, simplifies our proof production pipeline. Specifically, we can remove the previously-required sequential preprocessing step, which exhaustively performs unit propagation on the input, because CaDiCaL is now able to handle this natively. As a consequence, we no longer need to produce and prepend a proof for this preprocessing, and our proof production now directly emits a valid proof for the input formula’s unsatisfiability.

Our original setup featured postprocessing where the inverted combined proof is un-inverted and syntactically transformed to feature a compact domain of IDs. This step was required because of relatively poor tool support for the kind of LRAT proofs our approach emits (albeit perfectly valid in principle). Specifically, lrat-check from the drat-trim toolbox is not able gracefully to handle large gaps between subsequent clause IDs. The more recent checker lrat-trim [37] operates on 32-bit IDs and is hence not suitable either. We believe that formally verified LRAT checkers [31, 47] come with similar practical pitfalls. Our work thus raises demand for a well-engineered verified LRAT checker that is robust, fast, and comes with little opportunity cost over less reliable tools. While devising *and* verifying such a tool is out of scope for the work at hand, we make a first step by introducing a fast (unverified) LRAT checker that uses a robust hash table to handle arbitrary gaps in between clause IDs and that can be configured to operate directly on the *compressed* and *inverted* proof. Using this checker, our pipeline involves three steps: solving the original formula, assembling a combined proof, and checking the combined proof. Disk I/O is consequently reduced to writing and reading the combined proof only *once*.^7^ Due to the proof’s reversal, this single buffering step remains a strict requirement unless we assume that the entire proof can fit into main memory.

Lastly, following a suggestion by Peter Sanders, we introduce the notion of *SABs* (*satisfying assignment boosters*). In a computation that is expected to yield an independently-verifiable proof of unsatisfiability, solvers are usually made compliant by allowing only proof-producing operations. On satisfiable instances, however, this overhead turns out to be in vain. A *SAB* is a solver thread in such a computation that does *not* produce proof information and, consequently, cannot export learned clauses. It can, however, *import* shared clauses and find satisfiable assignments (and sometimes faster than its proof-producing peers). We include a small number of SABs in our setup (1 in 38 solver threads), mainly running Lingeling’s YalSAT local search solver [5] and occasionally Kissat [10] with SAT presets. In the case where a SAB happens to find unsatisfiability, we discard this result, waiting for a proof-backed result instead.

### Setup

We test our updated setup on an HPC cluster named **HoreKa**. Each compute node we use features two Intel Xeon Platinum 8368 sockets, each with 38 physical cores (76 hardware threads), and 256 GB of RAM. Fast proof writing is enabled by a 960 GB NVMe SSD at each node. Nodes are connected via an InfiniBand interconnect.

We again use the 2022 competition instances and test three different configurations of the latest version of MallobSat: our latest parallel/distributed proof-producing approach including 1/38 SABs and with one sharing per second (as in our base evaluation); the very same configuration except for disabling LRAT output and proof production; and the best currently known configuration (equal parts of Kissat, CaDiCaL, and Lingeling, and two sharings per second). We run each configuration at one (76 cores) and 20 compute nodes (1520 cores)—roughly on the levels of the parallel and distributed scales in our base evaluation. We also test the latest version of Gimsatul, at only one socket (38 cores) and at both sockets at once (76 cores), using drat-trim for proof checking. In order to save computational resources, we limit running times to 300 s for solving and 1500 s for producing and checking a proof across all runs. Lastly, we run sequential solver KissatMABHyWalk for up to 22,800 s ($6\frac{1}{3}$ h, matching the CPU resources of a 300 s 76-core run) to compute speedups. We refrain from storing and checking the DRAT proofs emitted by KissatMABHyWalk, instead directing them to /dev/null and assuming that checking times are similar to solving times (as indicated by the 2022 competition data).

**Fig. 7:**
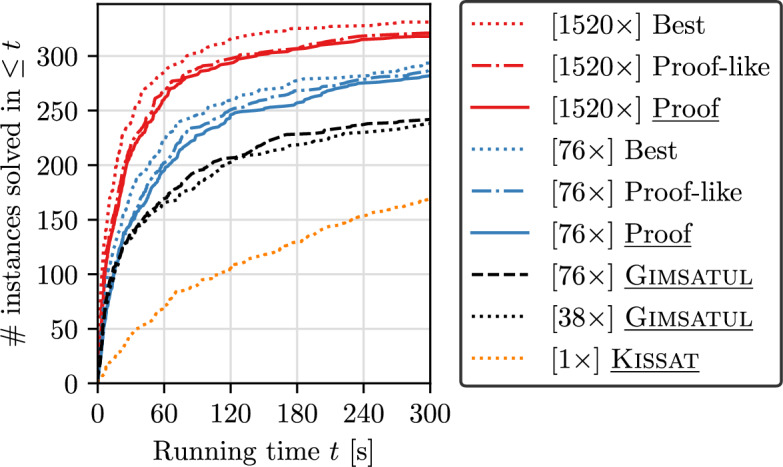
Pure solving times (excluding proof assembly and checking times; higher is better), where “Best” denotes the currently best performing configuration of MallobSat and “Proof-like” is equivalent to our proof-producing configuration (“Proof”) except that LRAT output and proof production themselves are kept disabled. Proof-producing approaches are underlined

### Results

As in Sect. 6, we first consider solving times only. Figure 7 and Table 6 show according results. In terms of absolute running times, the MallobSat system (“Best”) performs considerably better than MallobSat1600-KCLG in our original experiments (10.5% PAR-2 improvement and +6 solved instances at a consistent time limit of 300 s), notably on different hardware. More importantly, the margins between the best approach and our proof-producing approach have diminished. The respective mean slowdown of our approach is 38.7% at 76 cores and 53.8% at 1520 cores. Furthermore, we note that most of this overhead is not due to outputting proofs but rather due to the deviating solver and sharing configuration. Compared to the same configuration without emitting proof information (“Proof-like”), the slowdown of our approach is only 7.8% at 76 cores and 14.3% at 1520 cores. Gimsatul also performs better than in 2022 but is still not on par with MallobSat. Our SAB threads were the first and only threads to report satisfiability in 21 cases (both at 76 cores and at 1520 cores). This confirms that SABs can constitute effective accelerators for satisfiable instances, although further experiments would be needed to assess the opportunity cost in terms of UNSAT performance.

**Table 6:** Solver performance within 300 s of running time in terms of solved instances, PAR-2 score, and geom. mean speedup over KissatMABHyWalk w.r.t. commonly solved instances

Nodes	Solver	#	# SAT	# UNSAT	PAR-2	Speedup
seq.	KissatMABHyWalk	169	101	68	390.8	1.0
1	Gimsatul 38-core	238	128	110	275.8	6.6
	Gimsatul 76-core	241	132	109	268.6	7.4
	Proof	281	144	137	216.8	8.4
	Proof-like	287	147	140	207.7	8.7
	Best	293	149	144	194.9	10.8
20	Proof	318	156	162	152.2	17.5
	Proof-like	321	157	164	146.8	20.3
	Best	331	162	169	126.9	26.9

**Table 7:** Statistics on proof production and checking. Ratios are given as a multiple of the solving time. We list minima, maxima, medians, means, and the 10th and 90th percentiles—using the arithmetic mean for absolutes and the geometric mean for ratios

	Property		#	Min	p10	Med	Mean	p90	Max
Ratio	1 node asm		138	0.014	0.111	0.395	0.438	1.941	7.026
	1 node chk		138	0.010	0.051	0.422	0.367	1.849	6.534
	1 node asm+chk		138	0.066	0.249	0.783	0.870	3.587	13.560
	20 nodes asm		162	0.018	0.196	0.971	1.085	5.620	35.202
	20 nodes chk		157	0.019	0.130	2.489	1.681	10.155	86.608
	20 nodes asm+chk		157	0.148	0.412	3.584	2.906	15.872	121.810
	38-c. Gims. checking		68	0.272	1.283	7.057	12.186	159.859	279.848
Absolute	1-node proof size (GB)		138	0.000	0.065	0.801	2.764	9.132	49.533
	20-node proof size (GB)		163	0.000	0.214	3.126	11.645	30.957	233.880
	38-c. Gims. proof size (GB)		110	0.000	0.191	0.702	1.784	5.029	10.804
	1-node pruning factor		138	1.439	1.816	5.368	10.334	134.032	591.218
	20-node pruning factor		162	1.884	5.375	28.366	36.377	400.098	7124.047

**Fig. 8:**
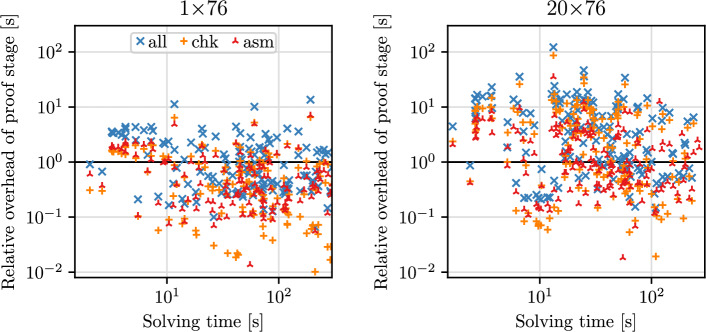
Overhead of proof-related stages (assembly, checking, and overall) relative to solving time, for our proof-producing approach at one and 20 nodes. Note the logarithmic scaling

Let us now discuss the relative overheads incurred by proof assembly and checking, shown in Table 7 and Fig. 8. The mean overhead incurred by proof assembly is 43.8% at 76 cores and 108% at 1520 cores—similar to the data in our base experiments (40.8% and 120% respectively). Checking overhead is reduced to 36.7% at 76 cores (from 47%) and 168% at 1520 cores (from 250%). Postprocessing is eliminated completely. As such, our large-scale distributed approach now only takes around 3$\times$ its own solving time to assemble and check a proof, while also achieving much better solving times in the first place. Generously assuming that checking KissatMABHyWalk’s proofs is exactly as fast as solving, the mean speedup of our proof-producing approach *including proof assembly and checking* is 11.5 at 76 cores and 15.5 at 1520 cores.

Our approach at 1520 cores produced five proofs that our LRAT checker was unable to check within 1500 s (25 min). The largest one among these is 234 GB in size^8^ and attempting to check it without imposing any limits resulted in an out-of-memory error on the machine with 256 GB of RAM after 1.6 h. The other four proofs, up to 179 GB in size, can be checked successfully in 28–34 min each.^9^ We believe that proofs of this size degenerate the Bloom filter we use for detecting duplicates, which results in missing deletion statements and thus higher memory requirements than necessary. We experimented with *exact* techniques for adding deletion statements but found them to degrade proof merging performance. Further research along this direction is needed, as is further improvement to our checker’s memory efficiency.

Since proof size and complexity increases with the number of solver threads (“rings”) of Gimsatul, we only gathered data for the more favorable 38-core configuration. Only 68 out of 110 complete proofs output by Gimsatul were checked successfully within 1500 s. Even for these 68 (relatively simple) checked proofs, the mean overhead of checking over solving is 1220%, which confirms the limited viability of using current DRAT checkers to validate proofs produced by Gimsatul [18].

## Conclusion

Distributed clause-sharing solvers are currently the fastest tools for solving a wide range of difficult SAT problems. However, their inability to produce proofs of unsatisfiability gravely impacts their trustworthiness and renders them unsuitable for critical applications. In the presented work, we examine mechanisms to add efficient support for proof generation to clause-sharing portfolio solvers. We introduce a distributed system with reasonable SAT solving performance that, in its current state, takes about three times its own solving time (at competitive solving performance) to assemble and check a proof of unsatisfiability based on partial proofs generated during solving. As such, our results demonstrate that it is feasible to make distributed clause-sharing solvers fully trustworthy and therefore viable for critical applications.

Following our research, it might be possible to generalize our approach to DRAT-based solvers by adding additional metadata, and this might allow easier retrofitting of the approach onto larger portfolios of solvers. Furthermore, it may be promising to investigate producing proofs in Mallob for the case where several MallobSat instances run concurrently and are rescaled dynamically [39] (cf. [43]). Other directions involve distributed combination and pruning algorithms for solvers that are not epoch-based.

## Data Availability

The experimental data and software associated with this work can be found at: 10.5281/zenodo.10184679.

## Footnotes

1 There are certain exceptions, such as *Monte Carlo* algorithms where providing some incorrect results is an explicit part of the specification.

2 https://doi.org/10.5281/zenodo.10184679.

3 https://github.com/marijnheule/drat-trim.

4 We use 2022 competition data, where Gimsatul [7] was still in an early stage of development (cf. [10]).

5 Throughout this discussion, we use the geometric mean if we refer to a mean of ratios.

6 Some of these improvements have been outlined in Ref. [43], forward-referencing the work at hand.

7 Our reverse file parser uses buffering to reverse-read roughly as fast as usual forward reading.

8 Belonging to cliquecoloring_n18_k6_c5.cnf.

9 When including these checking times, our approach’s mean total overhead increases from 2.91 to 3.02.
